# Editorial: Highlights in quality of life 2021/22

**DOI:** 10.3389/fgwh.2023.1149030

**Published:** 2023-05-26

**Authors:** Magdalena Emilia Grzybowska

**Affiliations:** Department of Gynecology, Obstetrics and Neonatology, Medical University of Gdańsk, Gdansk, Poland

**Keywords:** quality of life, cervical cancer, prevention, validation, database

**Editorial on the Research Topic** Highlights in quality of life 2021/22

Quality of life (QoL) has long been a part of medical research, attracting a steadily increasing amount of attention from scholars around the world. However, recent years have seen an unprecedented rise in research about the topic. Once the terms [(patient-reported outcomes) or (quality of life)] are entered into the search strategy at www.pubmed.gov, the growth immediately becomes apparent, with 2,038 papers published in 1992, 7,391 in 2002, but a staggering 40,740 in 2022 alone.

Patient-Reported Outcome Measures (PROs) are used to assess QoL. A PRO has been defined as any report about patient health status which comes directly from the patient, without being interpreted by a physician or anyone else (1). PROs are completed by patients to measure their perception of the functional well-being and health status (2). The questionnaires which are used to assess quality of life may be categorized into “generic” and “disease-specific”. Generic questionnaires allow for a more general assessment of the problem and can be applied to both, general and patient populations. Disease-specific questionnaires cover the issues which are specific to a particular disease entity or a population. They are also typically more responsive to change or treatment (2, 3). The use of any questionnaire in a given population and in a given language is only possible after a meticulously planned validation process. One of the first steps in the process of validation is translation and cultural adaptation of the instrument.

The paper *Protocol for the Cultural Translation and Adaptation of the World Endometriosis Research Foundation Endometriosis Phenome and Biobanking Harmonization Project Endometriosis Participant Questionnaire (EPHect)* presents the process in detail. Content and face validity of WERF EPHect EPQ-M were performed. The cross-cultural translation and adaptation of the questionnaire was carried out using the recommended guidelines and COnsensus-based Standards for the selection of health status Measurement Instruments (COSMIN standards) (4). The process was completed as follows: (1) conceptual equivalence, (2) forward translation, (3) backward translation, (4) expert panel revision, (5) cognitive testing, and (6) proofreading. The six phases of the cross-cultural adaptation are detailed in Figure 1 in the paper. The authors included the questionnaire in their Supplementary Material. According to the authors, endometriosis affects about 10% of the reproductive age women globally (190 million girls and women), remaining one of the most common causes of pelvic pain and infertility worldwide. Therefore, the development of tools for collecting non-surgical clinical and epidemiological data relevant to endometriosis research is critical to enable worldwide collaboration. According to the World Endometriosis Research Foundation, 54 institutions in 22 countries are registered as users of the tool (5).

International databases are becoming increasingly important, especially in the context of collecting data to conduct analyses on large groups of subjects in many conditions, e.g., in endometriosis, as presented in the paper. Research data registries contain shared datasets and facilitate collaborative research between various international centers. Surgical databases which collect information about the outcomes of the procedure and complications are of great importance.

The following article, *Community-Engaged Approaches to Cervical Cancer Prevention and Control in Sub-Saharan Africa: A Scoping Review,* deals with the issues of cervical cancer prevention. Organized screening has decreased cervical cancer incidence in the developed countries. However, at the same time, cervical cancer remains to be the leading cause of cancer-related mortality among African women. The paper is a narrative review describing community-engaged approaches to cervical cancer prevention and control methods in Sub-Saharan Africa (SSA). The Covidence systematic review software was used and nine articles were selected for the final analysis. The objectives of the review were as follows: to describe community engagement activities in cervical cancer prevention and control in SSA, to identify the aspects of cervical cancer research which used community engagement, and to describe the best practices for community engagement in achieving the goal of cervical cancer prevention and control. The study presented a Community-Based Participatory Research (CBPR) framework and analyzed the research, in accordance with its eight principles.

Cervical cancer is a global public health problem, with a particularly high burden in many low- and middle-income countries. The proven effectiveness of interventions such as vaccination against the most oncogenic human papillomavirus (HPV) types, and screening, particularly with HPV-based methods, makes cervical cancer a largely preventable disease (6). Substantial geographical and socioeconomic inequalities in cervical cancer have been reported. Twenty percent of all cases of cervical cancer globally were reported in Africa (6). Age-standardized incidence and morality rates in southern Africa were estimated at 36.4 (95% CI 35.8–37.1) and 20.6 (95% CI 20.1–21.1) per 100 000 woman-years, as compared to Western Europe 7.0 (95% CI 6.9–7.2) and 2.0 (95% CI 2.0–2.1), respectively (6). Public awareness and education projects to increase knowledge and generate more favorable attitudes toward HPV vaccinations are two of the most crucial steps in cervical cancer prevention (7). Little or no encouragement from the health providers results in low interest in preventive examinations. The situation might be improved by involving traditional and religious leaders, as well as the local media, to raise greater awareness among the population and to address public mistrust and concerns about HPV vaccination (8).

Both articles address the topic of research methodology. On the one hand, we have a paper on an international database covering a variety of patient medical data to improve our understanding of endometriosis. On the other hand, we have a paper evaluating the feasibility of community-based prevention of cervical cancer. The authors of these papers aim to reduce the morbidity of endometriosis and the incidence of cervical cancer. The WERF EPHect EPQ-M questionnaire, whose validation is presented by Mis et al., is released under the auspices of the World Endometriosis Research Foundation. The organization aims to be a global charity facilitating research into endometriosis to improve knowledge and treatment. In turn, the authors of the narrative review highlight the role of tribal leaders, religious leaders, and community health workers in increasing knowledge about cervical cancer and screening. They highlight strategies to identify and engage target populations and improve awareness.
